# Identification of novel target genes of nerve growth factor (NGF) in human mastocytoma cell line (HMC-1 (V560G c-Kit)) by transcriptome analysis

**DOI:** 10.1186/1471-2164-12-196

**Published:** 2011-04-18

**Authors:** Priyanka Dutta, Alexandra Koch, Bjoern Breyer, Heike Schneider, Oliver Dittrich-Breiholz, Michael Kracht, Teruko Tamura

**Affiliations:** 1 Institut fuer Biochemie, OE4310, Medizinische Hochschule Hannover, Carl-Neuberg-Str. 1, D-30623 Hannover, Germany; 2 Institut fuer Pharmakologie, Justus-Liebig-Universität Giessen, Frankfurt Str. 107, D-35392, Giessen, Germany

## Abstract

**Background:**

Nerve growth factor (NGF) is a potent growth factor that plays a key role in neuronal cell differentiation and may also play a role in hematopoietic differentiation. It has been shown that NGF induced synergistic action for the colony formation of CD34 positive hematopoietic progenitor cells treated with macrophage-colony stimulating factor (M-CSF or CSF-1), or stem cell factor (SCF). However, the exact role of NGF in hematopoietic system is unclear. It is also not clear whether NGF mediated signals in hematopoietic cells are identical to those in neuronal cells.

**Results:**

To study the signal transduction pathways induced by NGF treatment in hematopoietic cells, we utilized the mastocytoma cell line HMC-1(V560G c-Kit) which expresses the NGF receptor, tropomyosin-receptor-kinase (Trk)A, as well as the constitutively activated SCF receptor, V560G c-Kit, which can be inhibited completely by treatment with the potent tyrosine kinase inhibitor imatinib mesylate (imatinib). NGF rescues HMC-1(V560G c-Kit) cells from imatinib mediated cell death and promotes proliferation. To examine the NGF mediated proliferation and survival in these cells, we compared the NGF mediated upregulated genes (30 and 120 min after stimulation) to the downregulated genes by imatinib treatment (downregulation of c-Kit activity for 4 h) by transcriptome analysis. The following conclusions can be drawn from the microarray data: Firstly, gene expression profiling reveals 50% overlap of genes induced by NGF-TrkA with genes expressed downstream of V560G c-Kit. Secondly, NGF treatment does not enhance expression of genes involved in immune related functions that were down regulated by imatinib treatment. Thirdly, more than 55% of common upregulated genes are involved in cell proliferation and survival. Fourthly, we found Kruppel-like factor (KLF) 2 and Smad family member 7 (SMAD7) as the NGF mediated novel downstream genes in hematopoietic cells. Finally, the downregulation of KLF2 gene enhanced imatinib induced apoptosis.

**Conclusion:**

NGF does not induce genes which are involved in immune related functions, but induces proliferation and survival signals in HMC-1(V560G c-Kit) cells. Furthermore, the current data provide novel candidate genes, KLF2 and SMAD7 which are induced by NGF/TrkA activation in hematopoietic cells. Since the depletion of KLF2 causes enhanced apoptosis of HMC-1(V560G c-Kit), KLF2 may play a role in the NGF mediated survival signal.

## Background

Nerve growth factor (NGF) is a member of the family of neurotrophins and is essential for the survival and differentiation of neurons in central and peripheral nerve systems [1]. The binding of NGF to its high affinity receptor, tropomyosin-receptor-kinase(Trk)A, causes activation of the receptor associated tyrosine kinase and participates in the control of mitogenic, survival or differentiation pathways. It has been suggested that NGF and its receptor may also be involved in hematopoietic cell development [2,3]. In those studies NGF induced synergistic action for the colony formation of CD34 positive hematopoietic progenitor cells treated with the macrophage colony stimulating factor (M-CSF, or CSF-1) [3], or stem cell factor (SCF) [2]. However, the exact role of TrkA in hematopoietic cell differentiation remains unclear.

The receptor for SCF, c-Kit tyrosine kinase plays a key role in hematopoietic stem cell and mast cell survival, mitogenesis, proliferation, differentiation, adhesion, homing, migration, and functional activation. Despite diversity in the mechanisms of their activation by growth factor ligands [4], most receptor tyrosine kinases induce signals through the same pathways to typically enhance proliferation and prolong viability. These pathways include activation of the Ras/Raf/Erk, activation of signal transducers and activators of transcription (STATs), and phosphatidylinositol 3 kinase (PI3K). Indeed, c-Kit activation induces all of these pathways, while activated TrkA induces Ras/Raf/Erk, and PI3K pathways but does not cause tyrosine phosphorylation of endogenous STATs [5,6], suggesting that SCF and NGF not only induce common signal pathways, but also induce unique signal pathways. However, the differences between a set of genes which are upregulated by NGF and those upregulated by SCF in hematopoietic cells has not yet been studied.

The rat pheochromocytoma cell line, PC12, is one of the most thoroughly established systems to study the NGF mediated signal transduction pathway followed by neuronal differentiation. Various studies have investigated gene expression profiles in NGF-treated PC12 cells [7-14], however whether these upregulated genes are similar to genes in the hematopoietic system is not clear. Interestingly, leukemogenic mutant TrkA [5] does not induce tumor formation, but induces the differentiation of PC12 cells (Koch and Breyer, unpublished data), suggesting that NGF/TrkA signaling is different in neuronal and hematopoietic cells. We have previously shown that NGF-TrkA signaling partially rescues TrkA expressing Bcr-Abl transformed chronic myelogenous leukemia (CML) cells, such as K562, and Meg-01, from cell death induced by a potent inhibitor of Bcr-Abl tyrosine kinase, imatinib mesylate (imatinib). However, the effects of NGF on imatinib treated CML cells are modest. In the presence of NGF, the number of living K562 cells treated with imatinib increased by only 1.5-fold within 4 days and Meg-01 cells did not grow, but just survived for a longer period [15]. A dramatic effect of NGF treatment was observed in oncogenic c-Kit (V560G c-Kit) transformed human mastocytoma cells (HMC-1 (V560G c-Kit))[16] which are also induced to undergo apoptosis by treatment with imatinib. HMC-1 (V560G c-Kit) cells continue to grow nearly normally in the presence of both imatinib and NGF [15].

In this paper, using HMC-1 (V560G c-Kit) cells we compared NGF and SCF signaling in the same cell system. HMC-1 [16] expresses the activated SCF receptor, V560G and/or D816V c-Kit [17,18] and TrkA [19,20]. The kinase activity of V560G c-Kit can be inhibited completely by treatment with imatinib [21,22] and cells died within 3 days. NGF rescues HMC-1 (V560G c-Kit) cells from imatinib mediated cell death and promotes proliferation [15], indicating that NGF can take over mitogenic signaling in these cells. Therefore, we compared the NGF mediated upregulated genes (30 and 120 min after stimulation) to the downregulated genes by imatinib treatment (downregulation of c-Kit for 4 h) by transcriptome analysis. We found Kruppel-like factor (KLF) 2 and Smad family member 7 (SMAD7) as the NGF mediated novel down stream genes in hematopoietic cells and KLF2 may be involved in NGF mediated survival of imatinib treated cells.

## Results

### NGF rescues HMC-1 (V560G c-Kit) cells from imatinib mediated cell death and promotes proliferation

**Figure 1:**
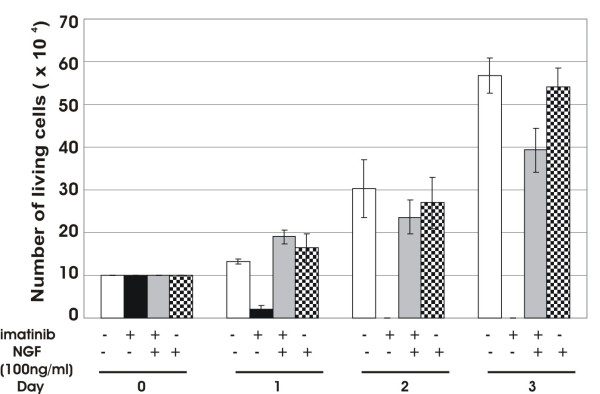
**NGF treatment rescues HMC-1 (V560G c-Kit) from imatinib induced rapid cell death**. Aliquots of 1 × 10^5 ^of HMC-1(V560G c-Kit) cells were grown in medium containing 10% FCS with or without NGF (100 ng/ml) in the presence or absence of imatinib (5 μM) for 3 days. Cells were stained with 0.1% Trypan Blue and living cells were counted using a Neubauer cell counting chamber. Cell numbers are presented as the mean ± S.D. for three independent experiments performed in triplicate.

To assess the biological effects of NGF on HMC-1(V560G c-Kit) cells in the absence of c-Kit-mediated signal, we treated the cells with 5 μM imatinib in the presence or absence of 100 ng/ml NGF. Viable cells were counted 1, 2, and 3 days after treatment using trypan blue cell exclusion assay. In agreement with previous data [15], more than 95% cell death was observed within 48 h of treatment with imatinib alone. In sharp contrast, HMC-1(V560G c-Kit) cells treated with imatinib in the presence of NGF continued to proliferate even after 72 h at a rate almost similar to untreated controls (Figure 1). In fact, NGF could support long-term survival (over 1 year) of these cells in the presence of imatinib (Koch and Dutta, unpublished data). In agreement with these data NGF treatment has been shown to induce mitogenic signals in CD34 positive hematopoietic progenitor cells [2]. Interestingly, it has been reported that the NGF stimulation induces the activation of Erk1/2 and PI3K, but does not induce tyrosine phosphorylation of STATs in PC12 cells [6], in promyeloid cell line 32D [5] or in HMC-1 cells (Koch and Dutta, unpublished data). On the other hand, STAT5 activation is required for the maintenance of mast cells [23], suggesting that NGF may induce unknown signals for the maintenance of HMC-1(V560G c-Kit) cells without STATs signaling. We next analyzed the gene profile induced by NGF treatment of HMC-1(V560G c-Kit) cells. To understand how NGF-TrkA activation counteracts the effect of c-Kit inhibition and promotes survival in HMC-1(V560G c-Kit) cells we performed gene expression profiling using a high density microarray technique employing the "Whole Human Genome Microarray" (G4112F, ID 014850, Agilent Technologies) that contains 45,015 probes. First, we determined the genes which were regulated as a result of c-Kit inhibition by comparing untreated HMC-1(V560G c-Kit) cells in serum free medium with cells after addition of 5 μM imatinib for 4 h. Second, we studied changes in gene expression caused by the addition of NGF (100 ng/ml) for 30 min (immediate early genes) and 2 h (delayed early genes) to the imatinib treated cells. Based on the filtering criteria mentioned in the methods section, 524 genes of known identities were downregulated and 328 genes were upregulated by treatment with imatinib, with expression ratios ranging from 2 to 45-fold and 2 to 10-fold, respectively. Twenty one genes of known identities were found induced after 30 min and 121 genes after 2 h of NGF stimulation following 4 h of imatinib treatment, with fold induction values ranging from 2 to 94- and 2 to 30-fold, respectively. Furthermore, NGF treatment repressed one gene after 30 min and seven genes after 2 h in imatinib treated cells.

NGF induced immediate and delayed early genes in imatinib treated HMC-1(V560G c-Kit) cells including several known NGF responsive immediate early genes such as the early growth response (EGR) family EGR1, 2 and 4, c-FOS, and JUNB being upregulated after 30 min of NGF treatment, followed by induction of delayed early genes such as NGFI-A binding protein 2 (NAB2), hairy and enhancer of split, (*Drosophila*) (HES1), Kruppel-like factor (KLF)10, and activating transcription factors 3 (ATF3) after 2 h.

**Figure 2:**
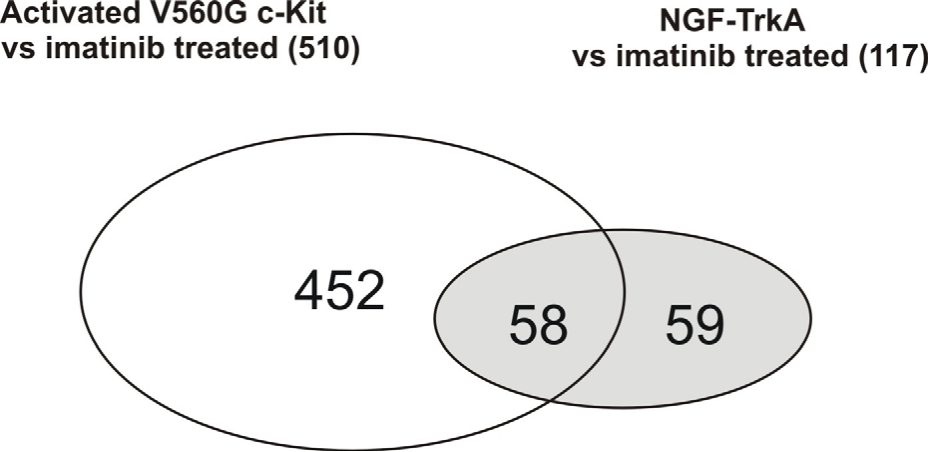
**Venn diagram of the number of genes which are upregulated by NGF treatment or downregulated by imatinib treatment**. Fifty eight genes from the two data sets overlap.

Prominently, EGR1 (or NGF1-A), first discovered as a NGF responsive gene in PC12 cells, was induced more than 90-fold within 30 min of TrkA activation providing an initial validation for our array [7]. To gain insight into the potential mechanism by which NGF can overcome imatinib induced apoptosis we focused our further investigation on the comparison of genes that were downregulated by imatinib and up-regulated by NGF treatment for 2 h. To derive a biological meaning from the individual lists of V560G c-Kit and NGF-TrkA-modulated genes, we uploaded these lists to Ingenuity Pathways Analysis (IPA, Ingenuity Systems, Mountain View, CA) application for biological function and pathway analysis. Out of a total of 524 genes modulated by imatinib treatment 510 genes mapped to the IPA knowledge database with 428 genes for functions/pathways analysis. Out of 117 NGF induced genes accepted by the IPA knowledge database (Table 1), 106 mapped for functions/pathways analysis. A comparison of the two gene lists revealed a common set of 58 genes out of 117 NGF upregulated genes (Table 1) that were also downmodulated by imatinib (Figure 2). This suggests that NGF-TrkA signaling regulates many genes the expression of which are also regulated by V560G c-Kit in HMC-1 cells.

**Table 1:** List of gene set regulated by NGF in HMC-1 (V560G c-Kit) cells.

Symbol	Gene accession ID	Description	Fold change
**CYTOKINES**			
CLCF1	NM_013246	cardiotrophin-like cytokine factor 1	2.6
FASLG	NM_000639	Fas ligand (TNF superfamily, member 6)	3.0
IL1B	NM_000576	interleukin 1, beta	2.2
LIF	NM_002309	leukemia inhibitory factor (cholinergic differentiation factor)	2.4
LTB	NM_002341	lymphotoxin beta (TNF superfamily, member 3)	2.2
**GROWTH FACTOR**			
GDF15	NM_004864	growth differentiation factor 15	3.2
**ENZYMES**			
ARL5B	NM_178815	ADP-ribosylation factor-like 5B	3.1
B3GALNT2	NM_152490	beta-1,3-N-acetylgalactosaminyltransferase 2	2.8
CYCS	NM_018947	cytochrome c, somatic	2.6
GALNT4	NM_003774	UDP-N-acetyl-alpha-D-galactosamine:polypeptide N-acetylgalactosaminyltransferase 4 (GalNAc-T4)	3.2
GEM	NM_005261	GTP binding protein overexpressed in skeletal muscle	2.9
LFNG	NM_001040167	LFNG O-fucosylpeptide 3-beta-N-acetylglucosaminyltransferase	2.5
PAICS	NM_006452	phosphoribosylaminoimidazole carboxylase, phosphoribosylaminoimidazole succinocarboxamide synthetase	2.9
RRAD	NM_004165	Ras-related associated with diabetes	2.5
TEP1	NM_007110	telomerase-associated protein 1	3.8
UBE2E1	NM_152653	ubiquitin-conjugating enzyme E2E 1 (UBC4/5 homolog, yeast)	4.2
**KINASES**			
AATK	AK131529	cDNA FLJ16758 fis, clone BRACE3038687, moderately similar to Homo sapiens apoptosis-associated tyrosine kinase (AATK)	2.5
PANK2	AL713654	Homo sapiens mRNA; cDNA DKFZp547J0513 (from clone DKFZp547J0513).	2.2
PLK3	NM_004073	polo-like kinase 3 (Drosophila)	5.1
PRKAR2A	NM_004157	protein kinase, cAMP-dependent, regulatory, type II, alpha	3.4
SGK1	NM_005627	serum/glucocorticoid regulated kinase	2.3
TRIB1	NM_025195	tribbles homolog 1 (Drosophila)	2.8
**LIGAND DEPENDENT NUCLEAR RECEPTOR**			
NR4A1	NM_002135	nuclear receptor subfamily 4, group A, member 1	4.7
NR4A2	NM_006186	nuclear receptor subfamily 4, group A, member 2	2.8
NR4A3	NM_006981	nuclear receptor subfamily 4, group A, member 3	2.6
**G-PROTEIN COUPLED RECEPTORS**			
CXCR4	NM_001008540	chemokine (C-X-C motif) receptor 4 (CXCR4), transcript variant 1	2.0
HRH1	NM_000861	histamine receptor H1	2.1
**PEPTIDASE**			
CTSZ	NM_001336	cathepsin Z	3.0
**TRANSCRIPTON REGULATOR**			
ACTN2	NM_001103	actinin, alpha 2	2.5
ARNTL2	NM_020183	aryl hydrocarbon receptor nuclear translocator-like 2	2.2
ATF3	NM_004024	Homo sapiens activating transcription factor 3 (ATF3), transcript variant 2	2.9
AXUD1	NM_033027	AXIN1 up-regulated 1 (AXUD1)	2.5
EGR1	NM_001964	early growth response 1	29.6
EGR2	NM_000399	early growth response 2	13.6
EGR3	NM_004430	early growth response 3	22.8
EGR4	NM_001965	early growth response 4	5.2
ETV5	NM_004454	ets variant 5	3.2
FOS	NM_005252	FBJ murine osteosarcoma viral oncogene homolog	3.5
FOSB	NM_006732	Homo sapiens FBJ murine osteosarcoma viral oncogene homolog B (FOSB)	3.7
FOSL1	NM_005438	FOS-like antigen 1	4.7
FOSL2	NM_005253	FOS-like antigen 2	2.5
HES1	NM_005524	hairy and enhancer of split 1, (Drosophila)	3.4
HOXB8	NM_024016	homeobox B8	14.1
JUNB	NM_002229	jun B proto-oncogene	2.1
KLF10	NM_005655	Kruppel-like factor 10 (KLF10), transcript variant 1	7.5
KLF2	NM_016270	Kruppel-like factor 2 (lung)	2.9
MAFF	NM_012323	v-maf musculoaponeurotic fibrosarcoma oncogene homolog F (avian) (MAFF), transcript variant 1	2.6
MYC	NM_002467	v-myc myelocytomatosis viral oncogene homolog (avian)	4.1
NAB2	NM_005967	NGFI-A binding protein 2 (EGR1 binding protein 2)	2.2
NOC2L	NM_015658	nucleolar complex associated 2 homolog (S. cerevisiae)	2.6
PBX2	NM_002586	pre-B-cell leukemia homeobox 2	2.7
POLR3E	AB040885	mRNA for KIAA1452 protein, partial cds.	4.0
RYBP	NM_012234	RING1 and YY1 binding protein	2.3
SF1	NM_004630	splicing factor 1	6.4
SKIL	NM_005414	SKI-like (SKIL)	3.1
SMAD7	NM_005904	SMAD family member 7	2.2
ST18	NM_014682	suppression of tumorigenicity 18 (breast carcinoma) (zinc finger protein)	2.0
ZFP36	NM_003407	zinc finger protein 36, C3H type, homolog (mouse)	2.3
ZFP36L1	NM_004928	zinc finger protein 36, C3H type-like 1	2.6
**TRANSPORTERS**			
AP1S1	NM_057089	adaptor-related protein complex 1, sigma 1 subunit (AP1S1), transcript variant 2	2.3
APOL6	NM_030641	apolipoprotein L, 6	3.9
GGA1	NM_001001560	golgi-associated, gamma adaptin ear containing, ARF binding protein 1	3.1
MCL1	NM_021960	myeloid cell leukemia sequence 1 (BCL2-related)	2.3
SLC2A14	BC060766	solute carrier family 2 (facilitated glucose transporter), member 14, mRNA (cDNA clone MGC:71510 IMAGE:5297510), complete cds. [BC060766]	2.9
SLC2A3	NM_006931	solute carrier family 2 (facilitated glucose transporter), member 3	2.5
STX1A	NM_004603	syntaxin 1A (brain) (STX1A)	2.5
TMC6	NM_152453	transmembrane and coiled-coil domains 5 (TMCO5)	3.3
**PHOSPHATASES**			
DUSP4	NM_001394	dual specificity phosphatase 4	2.7
DUSP5	NM_004419	dual specificity phosphatase 5	2.1
DUSP6	NM_001946	dual specificity phosphatase 6	19.9
EYA4	NM_004100	eyes absent homolog 4 (Drosophila)	2.1
**OTHERS**			
ADM	NM_001124	adrenomedullin	2.4
CASC5	NM_170589	cancer susceptibility candidate 5 (CASC5), transcript variant 1	3.3
CCDC71	NM_022903	coiled-coil domain containing 71	3.1
CCND1	NM_053056	cyclin D1	8.3
CDKN1A	NM_000389	cyclin-dependent kinase inhibitor 1A (p21, Cip1)	2.5
CLN8	NM_018941	ceroid-lipofuscinosis, neuronal 8 (epilepsy, progressive with mental retardation)	2.2
PSCD4	NM_013385	pleckstrin homology, Sec7 and coiled-coil domains 4 (PSCD4),	3.4
DDIT4	NM_019058	Homo sapiens DNA-damage-inducible transcript 4 (DDIT4),	2.4
FAM40B	NM_020704	family with sequence similarity 40, member B (FAM40B)	4.4
FBXL17	BC018548	F-box and leucine-rich repeat protein 17, mRNA (cDNA clone IMAGE:4215262), partial cds. [BC018548]	5.1
FST	NM_013409	follistatin	2.2
GNL3L	NM_019067	guanine nucleotide binding protein-like 3 (nucleolar)-like	2.3
GOLGA1	NM_002077	golgin A1	3.7
HNRNPA0	NM_006805	heterogeneous nuclear ribonucleoprotein A0 (HNRPA0)	3.0
IER2	NM_004907	immediate early response 2	2.5
IER3	NM_003897	immediate early response 3	9.2
LMNB1	NM_005573	lamin B1	3.0
LRRC8B	NM_015350	leucine rich repeat containing 8 family, member B (LRRC8B)	2.0
LUZP1	NM_033631	leucine zipper protein 1 (LUZP1)	2.8
MALAT1	NR_002819	metastasis associated lung adenocarcinoma transcript 1 (non-protein coding)	4.9
MED18	NM_017638	mediator of RNA polymerase II transcription, subunit 18 homolog (S. cerevisiae) (MED18)	15.0
METTL7A	NM_014033	methyltransferase like 7A	2.6
MYEOV	NM_138768	myeloma overexpressed (in a subset of t(11;14) positive multiple myelomas)	3.7
NUP188	BC005407	nucleoporin 188kDa, mRNA (cDNA clone IMAGE:3461492), partial cds. [BC005407]	2.8
PER2	NM_022817	period homolog 2 (Drosophila)	2.5
PHLDA1	NM_007350	pleckstrin homology-like domain, family A, member 1	9.7
PHLDA2	NM_003311	pleckstrin homology-like domain, family A, member 2	2.3
PMAIP1	NM_021127	phorbol-12-myristate-13-acetate-induced protein 1	3.6
PRNP	NM_000311	prion protein	3.2
RAPGEF5	D87467	mRNA for KIAA0277 gene, partial cds. [D87467]	18.5
RNF125	NM_017831	ring finger protein 125	5.1
RPL23	BC034378	ribosomal protein L23, mRNA (cDNA clone MGC:34067 IMAGE:5186030), complete cds. [BC034378]	2.3
SERPINE1	NM_000602	serpin peptidase inhibitor, clade E (nexin, plasminogen activator inhibitor type 1), member 1	3.3
SH2B3	NM_005475	SH2B adaptor protein 3	2.4
SH2D2A	NM_003975	SH2 domain protein 2A (SH2D2A)	2.1
SPRED1	NM_152594	sprouty-related, EVH1 domain containing 1	6.4
SPRED2	NM_181784	sprouty-related, EVH1 domain containing 2	3.1
SPRY2	NM_005842	sprouty homolog 2 (Drosophila)	6.6
TMEM49	NM_030938	transmembrane protein 49	7.3
TMEPAI	NM_020182	transmembrane, prostate androgen induced RNA (TMEPAI), transcript variant 1	4.2
TNFRSF12A	NM_016639	tumor necrosis factor receptor superfamily, member 12A	4.8
UBQLN1	NM_013438	ubiquilin 1	4.6
ZC3H7B	NM_017590	zinc finger CCCH-type containing 7B	3.9
ZCCHC2	NM_017742	zinc finger, CCHC domain containing 2	2.2
ZNF295	NM_020727	zinc finger protein 295 (ZNF295)	2.8
VIL2	NM_003379	villin 2 (ezrin) (VIL2)	2.3

### NGF-TrkA activation does not enhance expression of genes involved in immune related functions that were downregulated by imatinib treatment

Since the expression of 452 genes out of 510 genes which were downregulated by imatinib treatment for 4 h was not restored by the stimulation with NGF for 2 h (Figure 2), we next analyzed the two datasets using PANTHER (Protein ANalysis THrough Evolutionary Relationships) protein class analysis which measures the significance of certain functional categories among these targets by their enrichment relative to the total numbers in their respective categories. As shown in Table 2, immune response component genes such as cytokines and cytokine receptors genes were significantly downregulated (p = 2.45E-06 and 6.65E-04, respectively) by imatinib treatment. In contrast, NGF-TrkA does not activate receptor genes significantly (Table 2, NS). In addition, NGF-TrkA activated cytokine genes (p = 3.33E-03) but drastically fewer genes than c-Kit mediated gene modulation (Table 2). These data suggest that NGF can take over the proliferation signal but not immune related function induced by c-Kit.

**Table 2:** PANTHER analyses of c-Kit (downregulated genes by imatinib treatment) versus NGF-regulated genes which are involved in immune related function in HMC-1 (V560G c-Kit) cells (selected).

PANTHER Protein Class	V560G c-KIT (P-value)	NGF-TrkA (P-value)
**Cytokine**	2.45E-06	3.33E-03
**Cytokine/**interleukin superfamily	6.74E-05	2.15E-02
**Receptor**	1.17E-04	NS
**Receptor/**cytokine receptor	6.65E-04	NS
**receptor **/type I cytokine receptor	3.81E-03	NS
**Esterase**	2.10E-03	NS

NS: not significant

### More than 67% of NGF-TrkA upregulated genes are involved in cell survival and proliferation

**Figure 3:**
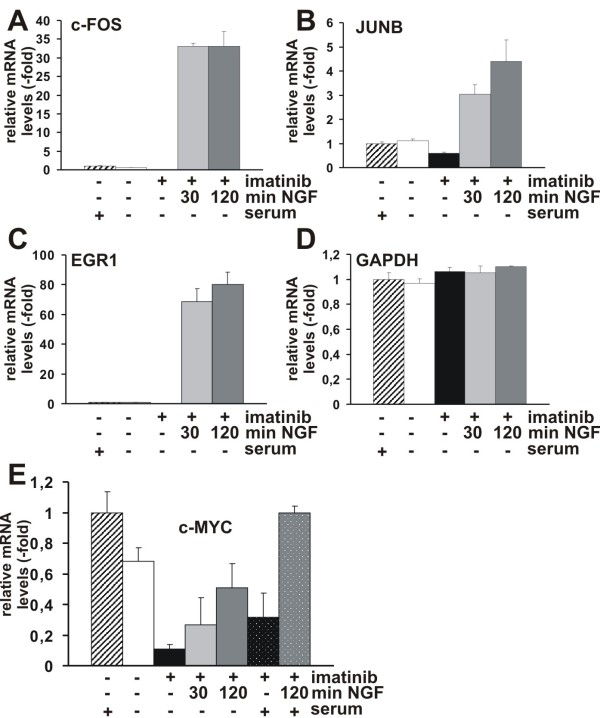
**Quantitative RT-PCR analysis of c-FOS, JUNB, EGR1 and c-MYC induced by NGF in HMC-1 (V560G c-Kit) cells**. HMC-1 (V560G c-Kit) cells were either incubated with serum or in serum free medium for 17 h, then treated without or with imatinib (5 μM) for 4 h prior to stimulation without or with NGF (100 ng/ml) for 30 and 120 min. Total RNA was isolated and, after reverse transcription, was examined by qRT-PCR using (A) c-FOS, (B) JUNB, (C) EGR1, (D) GAPDH and (E) c-MYC specific probes. Differences in mRNA expression levels are depicted as fold change values. Expression levels, in each case relative to GUSB as housekeeping gene, were normalized to gene expression in HMC-1 (V560G c-Kit) cells grown in serum containing medium. GAPDH served as a non-regulated control gene. Average values from three independent PCR reactions + standard error of the mean (SEM) are shown.

We next analyzed genes which are upregulated by NGF-TrkA signaling by IPA analysis. Significantly, over 67% of upregulated genes are involved in survival and proliferation (Table 3). Although the immediate-early response genes, such as EGR4, c-FOS, or FOSB are also downstream of c-Kit signaling, these genes are not constitutively expressing. Therefore, several c-Kit inducible genes were not downregulated by imatinib treatment. To confirm the micro array data, we performed quantitative reverse-transcriptase polymerase chain reaction (qRT-PCR) to examine the relative expression level of c-FOS, JUNB, EGR1, and c-MYC. HMC-1(V560G c-Kit) cells were incubated without serum for 17 h and were then treated with imatinib (5 μM) for 4 h. Cells were stimulated with NGF for 30 and 120 min. All samples were standardized by expression level of glucuronidase beta (GUSB) mRNA. In agreement with the micro array data, qRT-PCR analysis revealed that immediate-early response genes, such as c-FOS (Figure 3A, 33 fold) JUNB (Figure 3B, 3-4.4 fold), and EGR1 (Figure 3C, 68-80 fold), were upregulated upon stimulation with NGF compared to expression level in untreated HMC-1(V560G c-Kit) cells. Standardization of RNA level using a GUSB probe was confirmed by a GAPDH probe. The relative expression level of each sample was comparable (Figure 3D). c-MYC expression was also upregulated upon stimulation with NGF in imatinib treated cells in the absence of serum, however, its expression level was lower than that in imatinib-untreated cells with serum (Figure 3E). To examine whether high c-MYC expression in untreated cells is due to the activated c-Kit kinase and/or serum which may contain activation factor(s) of the c-MYC gene, we performed c-MYC specific qRT-PCR in the presence of serum with imatinib and/or NGF (120 min). Imatinib suppressed c-MYC expression about 70% even in the presence of serum, suggesting that activated c-Kit (V560G) induces c-MYC expression. However, in the presence of serum, NGF induces c-MYC expression 2-fold more than in the absence of serum, suggesting that serum and c-Kit or TrkA tyrosine kinase synergistically induce c-MYC expression (Figure 3E).

**Table 3:** Ingenuity biological function analyses of NGF regulated genes in HMC-1 (V560G c-Kit) cells (selected).

*CATEGORY* *sub category or function annotation*	*P-value*	*NGF induced molecules*
**1. CELL DEATH**		
Apoptosis	1.71E-15	AATK, ADM, ATF3, CCND1, CDKN1A, CLCF1, CLN8, CXCR4, CYCS, DDIT4, DUSP4, DUSP6, EGR1, EGR2, EGR3, EGR4, EZR, FASLG, FOS, FOSB, FOSL1, FST, GDF15, HES1, IER3, IL1B, KLF2, KLF10, LIF, LMNB1, LTB, MCL1, MYC, NOC2L, NR4A1, NR4A2, NR4A3, PHLDA1, PHLDA2, PLK3, PMAIP1, PMEPA1, PRNP, RYBP, SERPINE1, SGK1, SKIL, SLC2A3, SMAD7, SPRY2, STX1A, TNFRSF12A, TRIB1, ZFP36
Cell survival	1.61E-04	CCND1, CDKN1A, CLCF1, DUSP5, DUSP6, EGR3, FASLG, GDF15, HES1, IL1B, LIF, MCL1, MYC, NR4A1, PRNP, SERPINE1, SGK1, ZFP36
**2. CELL GROWTH AND PROLIFERATION**		
Growth	4.16E-12	ADM, ARNTL2, ATF3, CCND1, CDKN1A, CXCR4, DUSP5, DUSP6, EGR1, EGR2, EZR, FASLG, FOS, FOSL1, FST, GDF15, GEM, GGA1, HNRNPA0, IER3, IL1B, JUNB, KLF2, KLF10, LIF, MAFF, MCL1, MYC, NR4A2, NR4A3, PHLDA1, PHLDA2, PMEPA1, PRNP, RRAD, SERPINE1, SF1, SGK1, SH2B3, SKIL, SMAD7, SPRY2, TMEM49, TNFRSF12A
Proliferation	3.46E-10	ADM, CCND1, CDKN1A, CLCF1, CTSZ, CXCR4, EGR1, EGR2, EGR3, FASLG, FOS, FOSB, FOSL1, FOSL2, FST, GDF15, HES1, HRH1, IER3, IL1B, JUNB, KLF2, KLF10, LIF, MYC, NAB2, NR4A1, NR4A3, PMAIP1, PRNP, SERPINE1, SF1, SH2B3, SH2D2A, SKIL, SMAD7, SPRED1, SPRY2, TNFRSF12A, TRIB1, ZFP36, ZFP36L1
**3. CELL DEVELOPMENT**		
Development of blood cells	1.24E-11	ADM, CCND1, CDKN1A, CLCF1, CXCR4, DUSP5, EGR1, EGR2, EGR3, EZR, FASLG, FOS, FOSL1, HRH1, IER3, IL1B, JUNB, KLF2, KLF10, LFNG, LIF, LTB, MYC, NR4A1, PRNP, SH2B3, SH2D2A, SMAD7, SPRED2, TNFRSF12A, ZFP36
Development of tumor cell lines	1.20E-10	ADM, ATF3, CCND1, CDKN1A, CXCR4, DUSP5, EGR1, EGR2, FOS, FOSL1, FST, GDF15, GEM, HES1, IER3, IL1B, KLF2, LIF, MCL1, MYC, NAB2, NR4A2, PMEPA1, PRNP, RRAD, SERPINE1, SMAD7, SPRY2, TMEM49
Differentiation of cells	5.57E-09	ATF3, CCND1, CDKN1A, CLCF1, CXCR4, DUSP5, EGR1, EGR2, EGR3, FASLG, FOS, FOSL1, FOSL2, FST, HES1, HRH1, IL1B, JUNB, KLF10, LIF, MAFF, MCL1, MYC, NAB2, NR4A1, NR4A2, NR4A3, PRNP, SH2B3, SKIL, SMAD7, SPRED1, SPRED2, SPRY2, TNFRSF12A, ZFP36
Haematopoesis	2.82E-05	ATF3, CCND1, CDKN1A, CLCF1, CXCR4, DUSP5, EGR1, EGR2, EGR3, FASLG, FOS, FOSL1, FOSL2, FST, HES1, HRH1, IL1B, JUNB, KLF10, LIF, MAFF, MCL1, MYC, NAB2, NR4A1, NR4A2, NR4A3, PRNP, SH2B3, SKIL, SMAD7, SPRED1, SPRED2, SPRY2, TNFRSF12A, ZFP36
Maturation of cells	1.73E-05	CCND1, CDKN1A, EGR1, FASLG, FOS, HES1, IL1B, LFNG, LIF, MYC, NR4A2, PRNP
**4. CELL MORPHOLOGY**		
Morphology	8.65E-10	ADM, ATF3, CCND1, CDKN1A, EGR1, EZR, FOS, FOSL1, GDF15, GEM, JUNB, KLF2, LIF, MYC, PLK3, SERPINE1
Cell size	5.16E-04	CCND1, CDKN1A, KLF2, MYC
**5. CELLULAR FUNCTION AND MAINTAINANCE**		
T cell development	2.19E-09	CCND1, CDKN1A, CXCR4, EGR1, EGR2, EGR3, EZR, FASLG, FOS, IER3, IL1B, JUNB, KLF2, KLF10, LFNG, LIF, LTB, MYC, NR4A1, PRNP, SH2D2A, SMAD7
Cell death of T lymphocytes	8.90E-09	CDKN1A, CXCR4, EGR1, EGR3, EZR, FASLG, FOS, IER3, IL1B, KLF2, MYC, NR4A1, SH2D2A, SMAD7
**6. GENE EXPRESSION**		
Transcription	3.62E-08	ATF3, CCND1, CDKN1A, CSRNP1, DUSP4, EGR1, EGR2, EGR4, ETV5, FASLG, FOS, FOSB, FOSL1, FOSL2, FST, HES1, IL1B, JUNB, KLF2, KLF10, LIF, MAFF, MYC, NAB2, NOC2L, NR4A1, NR4A2, NR4A3, PBX2, PER2, RYBP, SGK1, SH2B3, SH2D2A, SKIL, SMAD7, ST18, ZFP36
Gene activation	7.79E-06	CCND1, EGR2, FOS, FOSB, FOSL1, FOSL2, KLF2, LIF, MYC, NR4A1, NR4A2
Gene repression	5.37E-05	FOS, FOSB, HES1, KLF10, MYC, NR4A2

Furthermore, 32 genes, including c-MYC, EGR1, EGR2, HES1, and KLF2 of 58 genes that were downmodulated by imatinib and upregulated upon stimulation with NGF are involved in survival and proliferation, suggesting that NGF/TrkA signaling may take over the survival and/or mitogenic signal in the imatinib treated HMC-1(V560G c-Kit) cells using these genes.

### Novel target genes, KLF2, and SMAD7 which were induced by NGF-TrkA signaling are involved in anti apoptosis signal in hematopoietic cell system

**Figure 4:**
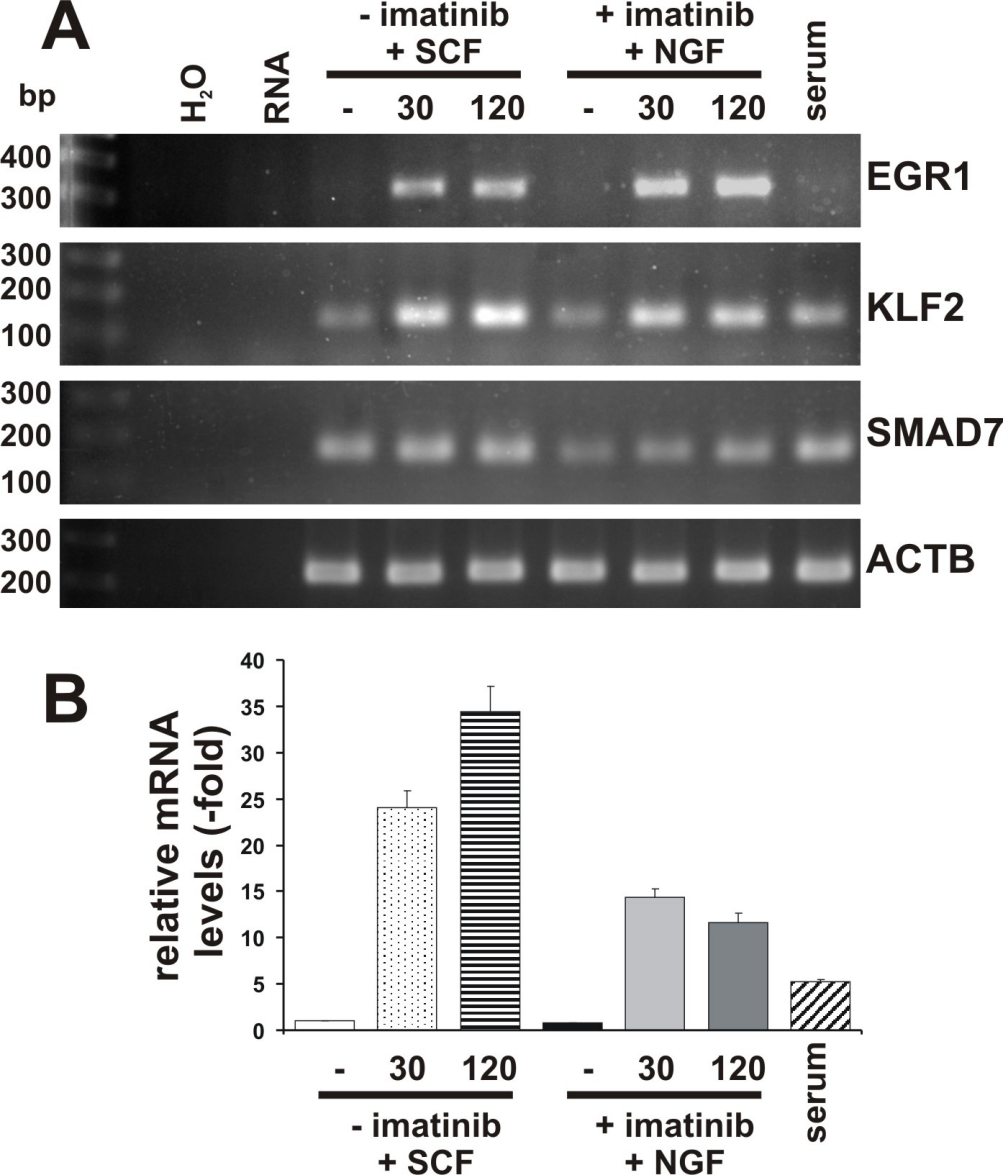
**KLF2 and SMAD7 genes were upregulated by stimulation with SCF or NGF**. A: HMC-1 (V560G c-Kit) cells were either incubated with serum (serum) or in serum free medium for 17 h, then treated without (-) or with (+) imatinib (5 μM) for 4 h prior to stimulation without (-) or with SCF (100 ng/ml) or NGF (100 ng/ml) for 30 (30) and 120 min (120). Total RNA was isolated and, after reverse transcription with Oligo dT-primers, cDNA was analyzed by semi-quantitative PCR for expression of EGR1, KLF2, SMAD7 and actin (ACTB). Water (H_2_O) and RNA (RNA) serve as negative controls. bp: base pairs of the DNA marker. B: Expression of KLF2 was quantified using TaqMan-PCR as in figure 3. Relative expression levels compared to GUSB were normalized to gene expression in serum-starved HMC-1(V560G c-Kit) cells. Average values from six independent PCR reactions + SEM are shown.

**Figure 5:**
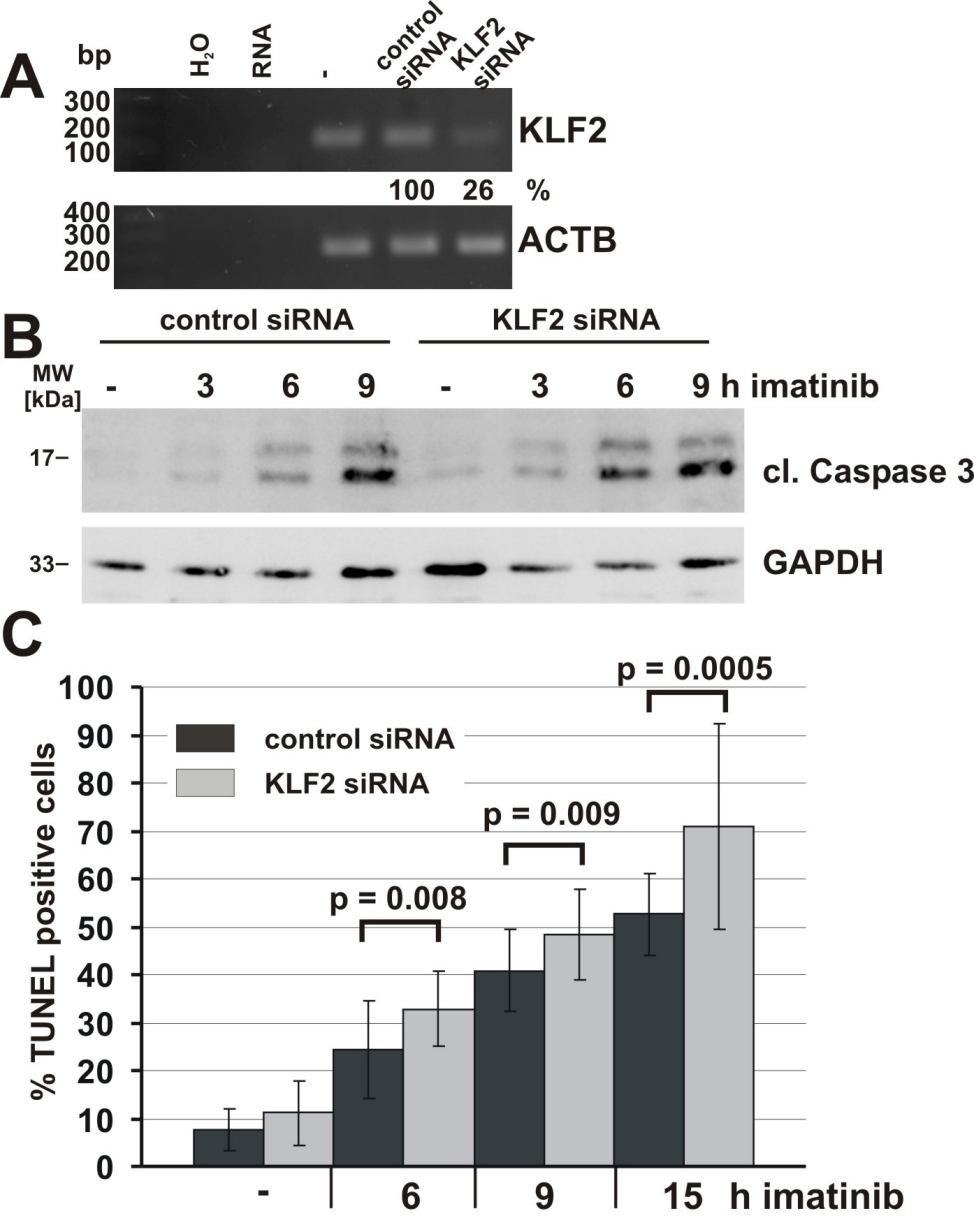
**Knockdown of KLF2 modulates the imatinib induced apoptosis**. A: HMC-1 cells were transfected with control siRNA or siRNA directed against KLF2 (KLF2 siRNA). 48 h after transfection, cDNA of untransfected (-) and transfected cells (control siRNA and KLF2 siRNA) was prepared and analyzed for expression of KLF2 and actin (ACTB) by semi-quantitative RT-PCR. KLF2 bands were quantified using TINA2.0 software and were normalized to actin. B: SiRNA transfected cells as in A were treated with imatinib (5 μM) for the indicated times. Every 3 h, whole cell lysates were prepared for detection of cleaved caspase-3 (cl. Caspase 3) and GAPDH by immunoblot. C: Cells were transfected and treated with imatinib as in B. At the indicated times after imatinib treatment, cells were fixed with paraformaldehyde and apoptotic cells were detected by TUNEL staining. Observations from different fields in the microscope (between 300 and 700 cells per experimental condition) are depicted as the mean +/- SD of % TUNEL positive cells. Student's *t*-test was applied to describe the significance of differences between cells treated with control siRNA and KLF2 siRNA. *P*-values ≤ 0.05 were considered as significant and are indicated above the corresponding columns. bp: base pairs of the DNA marker. MW: molecular weight.

Expression profiling of NGF-TrkA induced genes is well documented in neuronal cell systems [7-13]. However, there is no information about profiles of genes induced by NGF-TrkA signaling in a hematopoietic cell system. We therefore compared our upregulated genes to known NGF targets in neuronal cells. Several genes, such as the recently demonstrated ATF3, KLF10, and v-maf musculoaponeurotic fibrosarcoma oncogene family protein F (MAFF) [7] were found to be induced in our array. In addition to the above, we show for the first time the upregulation of potential novel TrkA target genes such as KLF2, SMAD7, and Homeobox members, HOXB8 and PBX2, upon NGF stimulation in HMC-1(V560G c-Kit) cells. Since it has been shown that an immediate early gene product, KLF2 activates SMAD7 expression [24], we examined the upregulation of KLF2, SMAD7 and EGR1 by RT-PCR (Figure 4). In agreement with array data, KLF2 was upregulated within 30 min similar to the EGR1 gene, however, SMAD7 was upregulated in 2 h (Figure 4A), suggesting that KLF2 may be the direct target gene of NGF/TrkA signaling, but not SMAD7. We next asked whether KLF2 and SMAD7 are targets of c-Kit signaling. Since oncogenic c-Kit (V560G) is not fully activated, SCF treatment is able to induce further upregulation of c-Kit mediated signaling [25]. HMC-1(V560G c-Kit) cells were grown in the absence of serum for 17 h, and were then stimulated with SCF (100ng/ml). The expression of KLF2, SMAD7 and EGR1 was then examined by RT-PCR. All three genes were upregulated by stimulation with SCF (Figure 4A). It should be noted that KLF2 and SMAD7 are also novel target genes for c-Kit signaling. To further confirm these data, we performed KLF2 specific qRT-PCR showing that serum starvation down-regulates KLF2 expression about 5-fold. However, upon stimulation with SCF or NGF in the absence of serum, within 30 min the KLF2 gene was upregulated 24-fold and 14-fold, respectively (Figure 4B). KLF2 is known to regulate self renewal and block the differentiation in embryo stem cells [26-28], suggesting that NGF/TrkA associates with a novel function other than neuronal differentiation. To examine whether KLF2 participates in the survival and proliferation signal induced by NGF, the KLF2 gene was downregulated by KLF2 specific siRNA in HMC-1(V560G c-Kit) cells. Two days after treatment of HMC-1 (V560G c-Kit) with KLF2 specific siRNA, the expression level of KLF2 declined to 26% (Figure 5A). The transient knockdown of KLF2 in HMC-1(V560G c-Kit) cells did not change the growth rate within 3 days after transfection under normal condition or in the presence of imatinib and NGF. We next examined whether KLF2 plays a role as a survival signal in imatinib treated HMC-1 (V560G c-Kit) cells. We began by examining caspase 3 cleavage. Cleaved caspase 3 was observed only 9 h after imatinib treatment in control siRNA treated cells, whereas in KLF2 specific siRNA treated cells caspase 3 was cleaved within 6 h (Figure 5B).

Furthermore, to assess the degree of apoptosis, sister culture cells were stained by an *in situ *cell death detection kit (Roche, Mannheim, Germany) for terminal deoxynucleotidyl transferase-mediated dUTP nick end labeling (TUNEL). In agreement with data obtained from caspase 3 cleavage, TUNEL positive cells appeared within 6 h after imatinib treatment in both KLF2 specific siRNA and control siRNA treated cells. However, numbers of TUNEL positive cells increased significantly faster in KLF2 siRNA treated cells than in control siRNA transfected cells 6 (p = 0.008), 9 (p = 0.009) and 15 h (p = 0.0005) after imatinib treatment (Figure 5C).

Since KLF2 specific siRNA transfectants still grow in the presence of NGF and imatinib, additional survival signals may be mediated by NGF treatment. However, our data strongly suggest that KLF2 is involved in an anti-apoptosis signal.

## Discussion

Cell differentiation and self-renewal are paralleled by a timely, ordered expression of a set of cytokines, growth factors and corresponding receptors. Many members of receptor tyrosine kinase family have emerged as key regulators of these critical cellular processes [4,29,30]. Humans have 58 known receptor tyrosine kinases, which fall into 20 subfamilies. Despite differences in structure, many of tyrosine kinases signal through the same pathways to typically enhance proliferation and prolong viability. These pathways include activation of the Ras/Raf/Erk, STATs and PI3K. These facts raised the question of whether each receptor tyrosine kinase is associated with a similar signaling potential, regulated by different expression patterns in different cell types, or whether each tyrosine kinase exhibits a unique signaling pathway.

It has previously been shown that TrkA and c-Kit are co-expressed in mast cells and hematopoietic CD34 positive cells. The treatment of CD34 positive cells with NGF showed the synergistic effects with the SCF treatment on colony formation. For mast cell culture *in vitro*, bone marrow cells are cultivated for 4-6 weeks in the presence of SCF, interleukin (IL) 3 and IL4 [31]. We examined whether mouse primary mast cells can survive in the presence of NGF, or NGF and IL3/IL4 in the absence of SCF. Under these conditions mouse mast cells did not survive in the absence of SCF. These data suggest that NGF does not assume the role of SCF in normal mast cells. According to PANTHER analysis [32,33], the difference of gene upregulation of cytokines, growth factors, and their receptors between SCF and NGF stimulation is significant, suggesting that upregulation of cytokines and their receptors play a role in survival of normal mast cells. In agreement with these data, few genes encoding cytokines/their receptors in PC12 cells were upregulated 24 h after NGF treatment [9], suggesting that NGF poorly induces cytokine and growth factor genes in different cell types.

It has been shown that STAT5 is required for c-Kit mediated mast cell survival and differentiation [23]. Although NGF does not induce tyrosine phosphorylation of STATs, HMC-1(V560G c-Kit) cells survive by NGF stimulation without c-Kit signaling. Thereby our array data provide novel candidate genes, KLF2, SMAD7, PBX2, and HOXB8 which are induced by NGF/TrkA activation in hematopoietic cells, and have not been reported as NGF target genes in the PC12 cell system [7-14]. On the other hand, another known target gene of NGF treatment in PC12 cells, wingless-related MMTV integration site 7B (Wnt7b) [14] was not upregulated by NGF treatment in HMC-1 (V560G c-Kit) cells, suggesting that Wnt7b may be a specific target gene for NGF signaling in neuronal cells. These data indicate that most NGF upregulated genes were common, but some of them may be cell-type specific. However, we cannot presently rule out the possibility that the difference of upregulated genes is due to differences between human (HMC-1) and rat (PC12) cells.

Interestingly, KLF2, SMAD7, PBX2, and HOXB8 are suggested to be involved in self-renewal or in anti differentiation signal of stem cells or hematopoietic stem cells [26,34-37]. We show here that KLF2 modulates imatinib-mediate apoptosis. Along the same line, it has been shown that KLF2-deficient T cells had a spontaneously activated phenotype and died rapidly from Fas-ligand-induced apoptosis [38], and induction of KLF2 expression corresponded with long-term T cell survival [39], suggesting that KLF2 plays a role in T cell survival. Furthermore, KLF2-/- embryos have a significantly increased number of primitive erythroid cells undergoing apoptotic cell death. These data suggest that the upregulation of the KLF2 gene induced by the stimulation with NGF plays a role in the survival signal in imatinib treated HMC-1(V560G c-Kit) cells.

## Conclusion

We compared the signaling of two structurally and functionally diverse receptor tyrosine kinases, c-Kit and TrkA, in hematopoietic cells. The c-Kit activation induces cytokines and their receptors, but TrkA does not, suggesting that the part of the signal pathways induced by the two receptors is different. However, TrkA is able to induce common novel downstream targets such as KLF2 and SMAD7 which has not been reported in the neuronal system, indicating that NGF induces genes which are involved in stem cell maintenance similar to c-Kit signaling in hematopoietic cells. Furthermore, upregulation of KLF2 may be involved in NGF mediated survival of imatinib treated cells.

## Methods

### Cell lines

HMC-1(V560G c-Kit) [16] were grown in RPMI1640 medium supplemented with 10-20% (v/v) fetal calf serum (FCS). The presence of V560G mutation and the absence of 816 mutation in c-Kit was confirmed by sequencing.

### Viability assay

HMC-1(V560G c-Kit) cells were grown in medium containing 10% FCS in the presence of 5 μM imatinib (kindly provided by Novartis, Basel, Switzerland) and/or 100 ng/ml human recombinant NGF (PeproTech Inc., Rocky Hill, NJ). Cells were counted in a Neubauer chamber using 0.1% Trypan Blue (Sigma-Aldrich Chemie GmbH, Steinheim, Germany).

### TUNEL assay

To assess the degree of apoptosis, an *in situ *cell death detection kit (Roche, Mannheim, Germany) was used for terminal deoxynucleotidyl transferase-mediated dUTP nick end labeling (TUNEL) staining.

### Growth factor stimulation, and RNA-isolation

Cells were serum starved for 17 h, then treated with dimethyl sulfoxide (DMSO) or 5 μM imatinib for 4 hours prior to stimulation with 100 ng/ml mouse recombinant SCF (PeproTech Inc.) or NGF, respectively. After 30 or 120 min the stimulation was stopped in ice-cold PBS. RNA was isolated from growth factor treated or untreated HMC-1(V560G c-Kit) cells using RNeasy Mini kit (Qiagen, Hilden, Germany) according to the manufacturer's protocol. Residual DNA contamination was removed with DNAseI (Invitrogen GmbH, Darmstadt, Germany) according to the manufacturer's recommendations, and the RNA was again purified with RNeasy Mini kit (Qiagen).

### Microarray analysis

The "Whole Human Genome Microarray" (G4112F, ID 014850, Agilent Technologies) used in this study contained 45015 oligonucleotide probes covering the entire human transcriptome. cRNA-synthesis was performed with the "Low RNA Input Linear Amplification Kit PLUS, Two-Color" (#5188-5340, Agilent Technologies) as directed by the manufacturer. cRNA fragmentation, hybridization and washing steps were also performed exactly as recommended by the manufacturer "Two-Color Microarray-Based Gene Expression Analysis Protocol V5.5" (see http://www.agilent.com for details) except that 4 μg of each labeled cRNA were used for hybridization. Slides were scanned on the Agilent Micro Array Scanner G2505 B at two different PMT settings, namely 100% (default setting) and 5%, to increase the dynamic range of the measurements. Data extraction and normalization were performed with the "Feature Extraction Software V9.5.3.1" by using the recommended default extraction protocol file: GE2-v5_95_Feb07.xml. Only probes with allocated gene symbols and arithmetic mean intensity >50 for both channels were considered for further analysis. Genes with p value ≤ 0.0001 and fold induction ratio of ≥ 2 were considered significantly induced.

### Accession Numbers

The complete microarray data have been deposited in NCBI's Gene Expression Omnibus and are accessible through GEO series accession number GSE28045.

### Functional and gene ontology analysis

Categorization of genes according to protein class was done using PANTHER (Protein Analysis Through Evolutionary Relationships) Classification Systems [32]. For each protein class, PANTHER calculates the number of genes identified in that category in both the list of differentially regulated genes and a reference list containing all the probe sets present on the chip and compares these results using the binomial test to determine if there are more genes than expected in the differentially regulated list [33]. Over-representation is defined by p < 0.05. Functional Analysis identifying the biological functions that were most significant to the data set were carried out using Ingenuity Pathways Analysis (IPA) (Ingenuity Systems, Mountain View, CA). Right-tailed Fisher's exact test was used to calculate a p-value determining the probability that each biological function and/or disease assigned to that data set is due to chance alone.

### RT-PCR and qRT-PCR

Reverse transcription was carried out using oligo dT primers and the Omniscript reverse transcriptase kit (Qiagen) following the instructions provided. PCRs were set up according to the following profile: an initial denaturation step of 94°C for 2 min, repeating cycles of 94°C for 30 seconds, annealing temperature given for each primer pair for 1 minute, and 72°C for 1 minute. Following primer pairs were used: human EGR-1 (NM_001964) forward primer: 5'-CAGCAGTCCCATTTACTCAG-3', reverse primer: 5'-GACTGGTAGCTGGTATTG-3' (annealing temperature 56°C, product size 345 bp); human KLF2 (NM_016270) forward primer: 5'-CTACACCAAGAGTTCGCATCTG-3', reverse primer: 5'-CCGTGTGCTTTCGGTAGTG-3'(annealing temperature 57°C, product size 137 bp); human SMAD7 (NM_005904) forward primer: 5'-ACTCCAGATACCCGATGGATTT-3', reverse primer: 5'-CCTCCCAGTATGCCACCAC-3' (annealing temperature 57°C, product size 174 bp); human beta-actin (ACTB, NM_001101) forward primer: 5'-CCCAAGGCCAACCGCGAGAAGAT-3', reverse primer: 5'-GTCCCGGCCAGCCAGGTCCAG-3' (annealing temperature 66°C, product size 219 bp). Separation of the DNA fragments was carried out on 2% (w/v) agarose gels, stained with ethidium bromide (2 μg/ml) and photographed under UV light. Quantitative (TaqMan) RT-PCR was performed as previously described [40]. TaqMan probes (Applied Biosystems, assay-IDs as follows: Hs00152928_m1(EGR1); Hs00357891_s1(JUNB); Hs00170630_m1(FOS); Hs00153408_m1(MYC); Hs00360439_g1(KLF2); Hs99999905_m1(GAPDH); Hs99999908_m1(GUSB)) were used with TaqMan^® ^Fast Universal PCR Master Mix (2×) (Applied Biosystems).

### Transfection, RNA interference and immunoblotting

SiRNA against human LKLF (KLF2) and control siRNA was purchased from Santa Cruz Biotechnology (Santa Cruz, CA). 4 × 10^6 ^HMC-1(V560G c-Kit) cells were transfected with 200 pmol of siRNA using Amaxa^® ^Cell Line Nucleofector Kit L with program T-020 in an Amaxa^® ^Nucleofector^® ^II device according to the manufacturer's instructions. Two days after transfection, cells were treated with imatinib (5 μM) for up to 15 h. During imatinib treatment, aliquots were prepared for analysis by TUNEL staining or immunoblot.

For immunoblot analysis, whole cell lysates were prepared using 1 × SDS buffer (80 mM Tris/HCl, pH 6.8, 2% sodium dodecyl sulfate (SDS), 10% glycerol, 5% beta-mercaptoethanol, 0.01% bromphenole blue). Then, cell lysates were analyzed for cleavage fragments of caspase 3 by immunoblot analysis using a polyclonal antibody against cleaved caspase-3 (Asp175) (Cell Signaling Technology, Beverly, MA) or GAPDH (Santa Cruz Biotechnology) as described previously [15]. Knockdown of KLF2 was verified by semi-quantitative RT-PCR and quantitative analysis was performed using TINA2.0 software (Raytest Isotopenmessgeraete GmbH, Straubenhardt, Germany).

## Authors' contributions

PD isolated RNA, carried out cell culture, and analyzed microarray data. AK carried out cell culture, immunoblot, siRNA and RT-PCR. BB analyzed microarray data by IPA HS, and ODB generated the microarray and the qRT-PCR data, MK provided the microarray facility, TT participated in the design of the study, contributed to the data analysis, and wrote and finalized manuscript. All authors participated in the discussion and approved the final manuscript.
